# Fibroblast growth factor 21 reverses high‐fat diet‐induced impairment of vascular function via the anti‐oxidative pathway in ApoE knockout mice

**DOI:** 10.1111/jcmm.17273

**Published:** 2022-03-20

**Authors:** Wen‐Pin Huang, Chi‐Yu Chen, Tzu‐Wen Lin, Chin‐Sung Kuo, Hsin‐Lei Huang, Po‐Hsun Huang, Shing‐Jong Lin

**Affiliations:** ^1^ 38007 Division of Cardiology Cheng Hsin General Hospital Taipei Taiwan; ^2^ Cardiovascular Research Center National Yang Ming Chiao Tung University Taipei Taiwan; ^3^ Institute of Clinical Medicine National Yang Ming Chiao Tung University Taipei Taiwan; ^4^ 46615 Division of Endocrinology and Metabolism Department of Medicine Taipei Veterans General Hospital Taipei Taiwan; ^5^ 38028 National Taipei University of Nursing and Health Sciences Taipei Taiwan; ^6^ 46615 Division of Cardiology Taipei Veterans General Hospital Taipei Taiwan; ^7^ 46615 Department of Critical Care Medicine Taipei Veterans General Hospital Taipei Taiwan; ^8^ 46615 Department of Medical Research Taipei Veterans General Hospital Taipei Taiwan; ^9^ Taipei Heart Institute Taipei Medical University Taipei Taiwan

**Keywords:** ApoE‐deficient mice, endothelial nitric oxide synthase, endothelial progenitor cell, fibroblast growth factor 21, vascular function

## Abstract

Circulating endothelial progenitor cells (EPCs), which function in vascular repair, are the markers of endothelial dysfunction and vascular health. Fibroblast growth factor 21 (FGF21), a liver‐secreted protein, plays a crucial role in glucose homeostasis and lipid metabolism. FGF21 has been reported to attenuate the progression of atherosclerosis, but its impact on EPCs under high oxidative stress conditions remains unclear. In vitro studies showed that the β‐klotho protein was expressed in cultured EPCs and that its expression was upregulated by FGF21 treatment. Hydrogen peroxide (H_2_O_2_)‐induced oxidative stress impaired EPC function, including cell viability, migration and tube formation. Pretreatment with FGF21 restored the functions of EPCs after the exposure to H_2_O_2_. Administration of N(ω)‐nitro‐L‐arginine methyl ester (L‐NAME), an inhibitor of nitric oxide synthase, inhibited the effects of FGF21 in alleviating oxidative injury by suppressing endothelial nitric oxide synthase (eNOS). In an in vivo study, the administration of FGF21 significantly reduced total cholesterol (TC) and blood glucose levels in apolipoprotein E (ApoE)‐deficient mice that were fed a high‐fat diet (HFD). Endothelial function, as reflected by acetylcholine‐stimulated aortic relaxation, was improved after FGF21 treatment in ApoE‐deficient mice. Analysis of mRNA levels in the aorta indicated that FGF21 increased the mRNA expression of eNOS and upregulated the expression of the antioxidant genes superoxide dismutase (SOD)1 and SOD2 in ApoE‐deficient mice. These data suggest that FGF21 improves EPC functions via the Akt/eNOS/nitric oxide (NO) pathway and reverses endothelial dysfunction under oxidative stress. Therefore, administration of FGF21 may ameliorate a HFD‐induced vascular injury in ApoE‐deficient mice.

## 1 INTRODUCTION

Obesity is independently associated with an increased rate of all‐cause mortality and is a risk factor for cardiovascular events. Excessive obesity‐related lipids accumulate in obese patients,^1^ exacerbate oxidative stress^2^ and impair endothelium‐dependent nitric oxide (NO)‐mediated vasodilation.^3^ Endothelial cells and endothelial progenitor cells (EPCs) are crucial in regulating vasodilation via endothelial nitric oxide synthase (eNOS)NO modulation.^4^, ^5^ EPC number has been recognized as a biomarker of cardiovascular disease (CVD). Circulating EPCs are essential in repairing vessel endothelial lesions and are responsible for neovascularization in ischaemic tissues.^6^ EPCs repair vascular injury by differentiating into mature endothelial cells and releasing cytokines to recruit EPCs to the injury area.^7^ Clinical studies have indicated that EPC function is impaired, and EPC number is decreased in patients with coronary artery disease (CAD).^8^ Moreover, high concentrations of hydrogen peroxide (H_2_O_2_) induce cellular apoptosis or senescence in EPCs.^9^

Fibroblast growth factor 21 (FGF21), which acts in an endocrine manner,^10^ is key in regulating glucose homeostasis, lipid metabolism and energy balance.^11^ Increased plasma levels of FGF21 were found to be positively related to type 2 diabetes (T2DM),^12^ obesity,^13^ and metabolic syndrome.^14^ In the cardiovascular system, secretion of FGF21 can protect the heart from hypertrophy, ischaemia‐reperfusion injury and oxidative stress.^15^ In addition to having cardioprotective functions, FGF21 has been shown to maintain vascular functions and exert an anti‐atherosclerotic effect.^16^ FGF21 treatment ameliorates H_2_O_2_‐induced apoptosis and cytotoxicity in human umbilical vein endothelial cells (HUVECs).^17^ Activation of FGF21 by binding to FGF receptor (FGFRs) complexed with the essential co‐receptor β‐klotho was shown to modulate diverse anti‐atherosclerotic effects.^18^ However, the relationship between plasma FGF21 and circulating EPCs and the impact of FGF21 on high‐fat diet (HFD)‐induced endothelial dysfunction remain unclear. We therefore designed this study to investigate the effects of FGF21 on cultured EPCs under H_2_O_2_‐induced high oxidative stress conditions and assess the potential impact of FGF21 on endothelial dysfunction in hypercholesterolaemic mice.

## 2 MATERIALS AND METHODS

### 2.1 Isolation and cultivation of EPCs

EPCs were isolated from peripheral blood mononuclear cells (MNCs) of healthy young adult volunteers as previously described.^19^ Briefly, peripheral blood MNCs isolated by Histopaque‐1077 (1.077 g/ml; Sigma‐Aldrich, USA) density‐gradient centrifugation to minimize cellular blood components such as platelets. 1 × 10^7^ MNCs were plated in endothelial growth medium‐2 (EGM‐2; Lonza Ltd., Basel, Switzerland) with supplementation (hydrocortisone, hFGF‐B, VEGF, R3‐IGF‐1, ascorbic acid, hEGF, GA‐1000 and 10% foetal bovine serum) in a fibronectin‐coated 6‐well plate at 5% CO_2_, 37°C. The medium changed every two days, and colonies of EPCs appeared after 2–3 weeks. EPC colonies were cultured on fibronectin‐coated plates and used at the passage 3 to 6 for further experiments. The EPCs exhibited ‘cobblestone’ morphology and a monolayer growth pattern that is typical of mature endothelial cells at confluence. EPCs were characterized by immunofluorescence staining against VE‐cadherin, CD31, CD34, KDR, CD133 and eNOS (Figure S1).

### 2.2 Measurement of ROS production

Intracellular ROS levels were measured by a Fluorometric Intracellular ROS Kit (MAK142; Sigma‐Aldrich, USA). EPCs were seeded on fibronectin‐coated 12 mm cover glasses in a 24‐well plate. The EPCs were treated with the indicated concentration of FGF21 for 12 h and 600 µM H_2_O_2_ for 1 h. The cells were washed with PBS and incubated with ROS Detection Reagent at 5% CO_2_ and 37°C for 1 h. After 1 h, the samples were carefully washed with PBS and then stained with DAPI (1:1000; ab228549; Abcam, USA) for 15 min at room temperature. The cover glasses were mounted with mounting medium (Dako, USA), and images were captured with a laser confocal microscope (ZEISS LSM 880, ZEISS, Germany).

### 2.3 Cell viability assay

Cell viability was analysed by the Cell Counting Kit‐8 (CK04; Dojindo Molecular Technologies, USA) assay. EPCs were seeded in a 48‐well plate. The cells were treated with FGF21 for 12 h and then exposed to 600 μM H_2_O_2_ for 10 . CCK‐8 solution was added to the medium, and the cells were incubated for 2 h at 5% CO_2_ and 37°C. Finally, the absorbance was measured at 450 nm using an ELISA reader.

### 2.4 Measurement of NO production

The level of NO was measured with a Nitric Oxide Colorimetric Assay Kit (K262; Biovision, USA). The medium was incubated with nitrate reductase for 1 h to convert nitrate into nitrite. Griess reagent was added after incubation, and the absorbance was measured at 540 nm. The nitrite concentration was calculated using a standard nitrite curve.

### 2.5 EPC tube formation assay

EPCs were seeded in a 6‐well plate and treated with the indicated concentration of FGF21 for 12 h and 600 µM H_2_O_2_ for 10 h. Tube formation was assessed with an In Vitro Angiogenesis Assay Kit (ECM625; Merck Millipore, USA). ECMatrix Gel was mixed with ECMatrix Diluent Buffer, and 50 µl of the mixture was added to each well of a precooled 96‐well culture plate. The 96‐well plate was incubated for 1 h at 37°C to allow the ECMatrix gel to solidify. EPCs (1 × 10^4^) were seeded in the 96‐well plate in EGM‐2 medium and incubated for 16 h at 37°C. EPC tube formation was evaluated by counting the tube number in five randomly chosen high‐power (X100) microscopic fields.

### 2.6 EPCs migration assay

EPC migration was assessed by a modified Boyden chamber assay (PSET010R5; Merck Millipore, USA). EPCs were treated with FGF21 for 2 h before treatment with H_2_O_2_. A total of 4 × 10^4^ cells were plated in 150 µl serum‐free EBM‐2 medium in the upper chamber. The lower chamber was filled with 500 µl medium containing 5% FBS. After 8 h of incubation, the cells in the chamber were washed with PBS and fixed with 2% paraformaldehyde for 15 min at 37°C. After washing with PBS, the cells were stained with haematoxylin for 10 min at room temperature. The degree of EPC migration was evaluated by counting migrated cells in six randomly chosen high‐power (X100) microscopic fields.

### 2.7 Animals

Male apolipoprotein E (ApoE) knockout (KO) mice on the C57BL/6 background were obtained from The Jackson Laboratory (B6.129P2‐Apoetm1Unc/J). The animals were kept in microisolator cages on a 12‐h day/night cycle with unrestricted access to water. Six‐week‐old male ApoE‐KO mice were randomly divided into two groups: (1) ApoE‐KO mice fed a HFD (5TJN; TestDiet 5342) and treated with saline (*n* = 7; ApoE‐KO + FHD) and (2) ApoE‐KO mice fed a HFD and treated with recombinant FGF21 (*n* = 7; ApoE‐KO + HFD + FGF21). Mice were fed the appropriate diet for 8 weeks. After feeding for 4 weeks, the mice were given saline or 0.1 mg/kg recombinant FGF21 (SRP4066; Sigma‐Aldrich, USA) daily by intraperitoneal injection for 4 weeks.

All experimental procedures and protocols involving animals were conducted in accordance with the institutional guidelines for animal care of National Yang Ming Chiao Tung University (Taipei, Taiwan; IACUC no. 2019‐089; Approval date: 2019‐July‐08) and the Guide for the Care and Use of Laboratory Animals of the US National Institutes of Health (8th edition, 2011). All methods in this study are reported in accordance with the ARRIVE guidelines.

### 2.8 Measurement of serum blood chemical parameters

After 12 h of fasting, blood samples were collected from the facial vein. Serum was obtained by centrifugation at 3000×g for 15 min at room temperature. Total cholesterol (TC), triglyceride (TG) and blood glucose levels were measured with an Automated Clinical Chemistry Analyzer (Fuji DRI‐chem 4000i; Fujifilm Corporation, Japan).

### 2.9 Measurement of vascular reactivity

As described in a previous study, we evaluated endothelial function by the aortic ring relaxation test.^5^ After 8 weeks of HFD feeding, the mice were anesthetized by intraperitoneal injection of 250 mg/kg avertin. Approximately 4 mm piece of the descending thoracic aorta was excised and placed in ice‐cold oxygenated Krebs bicarbonate buffer (118 mM NaCl, 4.7 mM KCl, 1.1 mM MgSO_4_, 1.2 mM KH_2_PO_4_, 25 mM NaHCO_3_, 1.5 mM CaCl_2_ and 5.6 mM glucose; pH 7.4) at 5% CO_2_ and 95% O_2_. The fat and connective tissue covering the surface of the aorta were carefully removed. The aorta was mounted on two steel hooks connected to a force‐displacement transducer (Model FT3E; Grass, West Warwick, RI, USA) and transferred to a chamber containing 10 ml of Krebs buffer. The aorta was equilibrated under 1‐G tension for 1 h at 37°C. Vascular reactivity was measured in aortic rings in which the endothelium was precontracted with different concentrations of phenylephrine (10^−9^–10^−5^ mol/L). After submaximal concentrations were reached, endothelium‐dependent relaxation was evaluated using an acetylcholine (10^−9^–10^−5^ mol/L) concentration‐response curve. Relaxation was calculated as the percentage of precontractile vascular tone.

### 2.10 RNA extraction and quantitative real‐time PCR

Total RNA was isolated from the mouse aorta with NucleoZOL (REF 740404.200; Macherey‐Nagel, Germany). Five hundred nanograms of total RNA were reverse‐transcribed into cDNA with a cDNA synthesis kit (K1621; Thermo Fisher Scientific, USA), and SYBR Green Mastermix (4309155; Thermo Fisher Scientific, USA) was used for real‐time PCR. The reaction and signal detection were performed on a StepOnePlusTM Real‐Time PCR System (Applied Biosystems, USA). The primer sequences were as follows: eNOS: forward‐TCAGCCATCACAGTGTTCCC, reverse‐ATAGCCCGCATAGCGTATCAG; superoxide dismutase (SOD)1: forward‐AACCAGTTGTGTTGTCAGGAC, reverse‐CCACCATGTTTCTTAGAGTGAGG; and SOD2: forward‐CAGACCTGCCTTACGACTATGG, reverse‐CTCGGTGGCGTTGAGATTGTT). GAPDH (forward‐AGGTCGGTGTGAACGGATTTG, reverse‐TGTAGACCATGTAGTTGAGGTCA) was used as an internal control.

### 2.11 Immunofluorescence

EPCs were seeded on fibronectin‐coated 12 mm cover glasses in 24‐well plates. After FGF21 treatment, the cells were washed twice with PBS and fixed with 2% paraformaldehyde for 15 min at 37°C. The cells were incubated with 10% BSA (A7906, Sigma‐Aldrich, USA) for 1 h at room temperature to block nonspecific binding. After blocking, the cells were incubated with primary antibody against β‐klotho (1:20; AF5889; R&D Systems, USA) for 2 h at room temperature and then incubated with FITC‐conjugated secondary antibody for 1 h at room temperature. After washing with PBS, the cells were stained with DAPI (1:1000; ab228549; Abcam, USA) for 15 min at room temperature. The cover glasses were mounted with mounting medium (Dako, USA), and images were captured with a laser confocal microscope (ZEISS LSM 880, ZEISS, Germany).

### 2.12 Western blotting

EPCs were washed with cold PBS and lysed with protein lysis buffer (62.5 mM Tris‐HCl, 2% SDS, 10% glycerol, 1 mM PMSF and 1 μg/ml aprotinin, pepstatin and leupeptin). The protein concentration was determined by the Bradford protein assay (#5000006, Bio‐Rad, USA). The proteins were separated by SDS‐PAGE and then transferred onto a PVDF membrane by using iBlot™ Transfer Stacks (Invitrogen, USA). The membrane was blocked with 3% BSA (A7906, Sigma‐Aldrich, USA) in TBST for 1 h at room temperature and then incubated with primary antibodies against phosphorylated eNOS (*p*‐eNOS) (Ser1177) (1:1000; #9571; Cell Signaling Technology, USA), eNOS (1:1000; 07‐520; Merck Millipore, USA), phosphorylated Akt (*p*‐Akt) (Ser473) (1:1000; #9271; Cell Signaling Technology, USA), Akt (1:1000; #9272; Cell Signaling Technology, USA) and β‐actin (1:5000; A5316; Sigma‐Aldrich, USA) overnight at 4°C. After washing 2 times with TBST for 10 min, the membrane was incubated with rabbit, mouse, or goat secondary antibody for 1 h at room temperature. After washing 2 times with TBST for 10 min, the signals were detected with chemiluminescence detection reagents (#NEL121001EA, PerkinElmer, USA).

### 2.13 Statistical analysis

The data are expressed as the means ± standard errors of the mean. Comparisons between two groups were performed using unpaired Student's *t* test, and multiple group comparisons were performed using one‐way analysis of variance followed by Scheffe's multiple comparison post hoc test. The analyses were conducted using SPSS software (version 14; SPSS, Chicago, IL, USA). *p* values < 0.05 were considered statistically significant.

## 3 RESULTS

### 3.1 The expression of β‐klotho in EPCs

**FIGURE 1:**
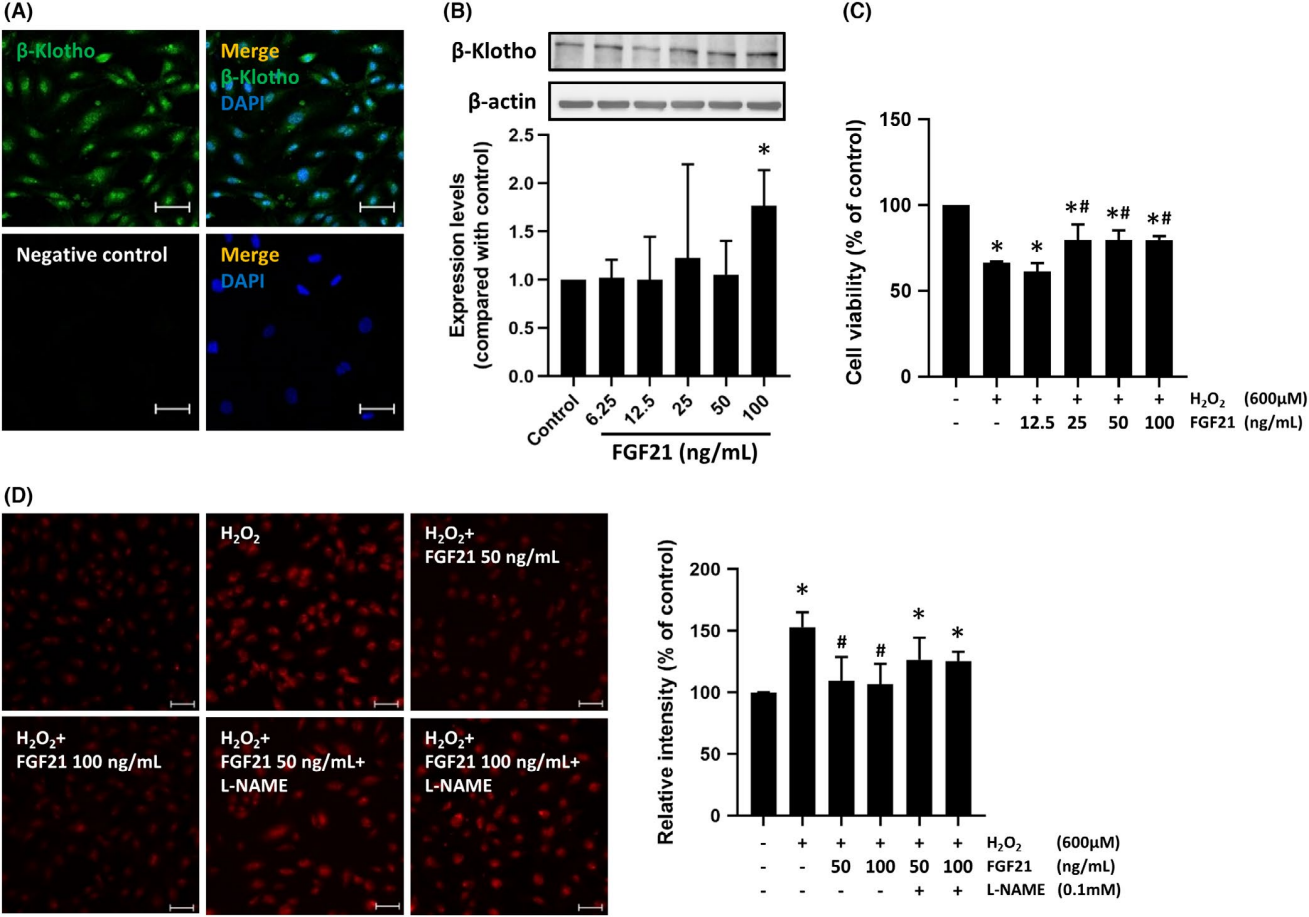
Effects of FGF21 on β‐klotho expression and oxidative stress in EPCs. After FGF21 treatment, cells were (A) stained with an β‐klotho antibody, and (B) protein expression was analysed by Western blotting. (C) The effects of FGF21 on EPC viability were analysed by the CCK‐8 assay. (D) EPCs were treated with the indicated concentration of FGF21 for 12 h and 600 µM H_2_O_2_ for 1 h. Intracellular ROS levels were measured using a Fluorometric Intracellular ROS Kit. The results are expressed as the mean ± SEM of five separate experiments run in triplicate (*p < 0.01 vs. control, #*p* < 0.01 vs. H_2_O_2_ only)

β‐klotho is an essential component of the FGF21 receptor. To investigate whether β‐klotho was expressed in EPCs, we assessed the expression of β‐klotho by immunofluorescence staining and Western blotting. The results showed that β‐klotho was expressed in EPCs (Figure 1A). In additionally, treatment with FGF21 significantly increased the expression of β‐klotho (Figure 1B).

### 3.2 FGF21 ameliorated H_2_O_2_‐induced cell damage in EPCs

Endothelial dysfunction is the initial step in atherosclerosis and one of the causes of endothelial dysfunction is oxidative stress. We investigated the effects of FGF21 on cell viability under H_2_O_2_ exposure in EPCs. EPCs were pretreated with FGF21 for 12 h and then treated with H_2_O_2_ for another 10 h. Compared with control treatment, H_2_O_2_ decreased the viability of EPCs by 30%. Pretreatment with FGF21 significantly reversed the damage to EPCs in the presence of H_2_O_2_ (H_2_O_2_ vs. H_2_O_2_ + 50 ng FGF21 and H_2_O_2_ + 100 ng FGF21: 70.44 ± 3.57 vs. 79.81 ± 5.48 and 79.56 ± 2.41, respectively; *p* < 0.05) (Figure 1C).

### 3.3 FGF21 attenuated H_2_O_2_‐induced ROS production via eNOS in EPCs

Excessive or sustained ROS production might reduce eNOS activity and NO production. Therefore, H_2_O_2_ was used to mimic an oxidative stress environment. The results showed that FGF21 attenuated H_2_O_2_‐induced ROS production in EPCs. As shown in Figure 1D, ROS production significantly increased in EPCs exposed to H_2_O_2_ compared with control EPCs. Pretreatment with FGF21 for 12 h significantly attenuated ROS production in EPCs. The FGF21‐induced reduction in ROS production was reversed by the administration of N(ω)‐nitro‐L‐arginine methyl ester (L‐NAME), which is an eNOS inhibitor.

### 3.4 FGF21 prevented the H_2_O_2_‐induction impairment of EPC function via eNOS

**FIGURE 2:**
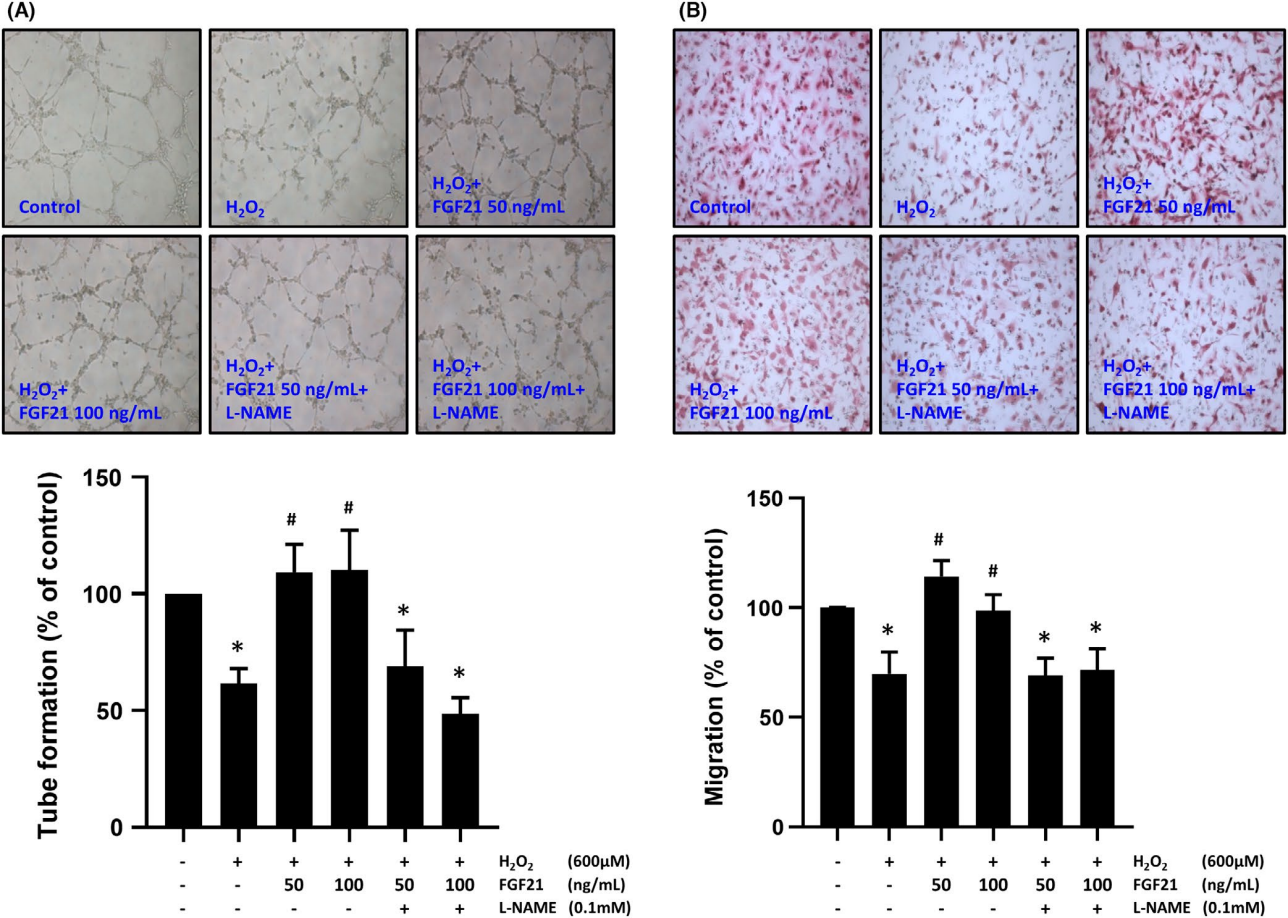
FGF21 rescued oxidative stress‐induced EPC dysfunction through the NO pathway. (A) EPCs were treated with the indicated concentration of FGF21 for 12 h and 600 µM H_2_O_2_ for 10 h. An in vitro angiogenesis assay was used to assess EPC tube formation ability. Representative photographs of in vitro angiogenesis are shown. The average total area of complete tubes formed by cells was compared using ImageJ software. (B) EPCs were treated with the indicated concentration of FGF21 for 12 h and 600 µM H_2_O_2_ for 10 h and placed in the upper chamber of a Transwell system to allow migration toward FBS (5% FBS was placed in the lower chamber). The histogram shows the percentage of migrating cells. These results are expressed as the mean ± SEM of five separate experiments run in triplicate (*p < 0.01 vs. control, #*p* < 0.01 vs. H_2_O_2_ only)

We further investigated whether FGF21 improves the functions of EPCs in tube formation and migration under H_2_O_2_ exposure. We used an in vitro angiogenesis assay to evaluate tube formation ability. Exposure to H_2_O_2_ significantly inhibited the tube formation ability of EPCs, while treatment with FGF21 increased the tube formation ability of EPCs (Figure 2A). Similar results were observed in the Boyden chamber assay. Treatment with H_2_O_2_ reduced the migration ability of EPCs, whereas administration of FGF21 improved the migration ability of EPCs under H_2_O_2_ exposure (Figure 2B). Administration of L‐NAME inhibited the beneficial effects of FGF21 on the tube formation and migration ability of EPCs (Figure 2A, B). These data suggest that treatment with FGF21 significantly improved EPC functions under high oxidative stress conditions.

### 3.5 FGF21 increased the expression of p‐eNOS and p‐Akt in EPCs, which was suppressed by H_2_O_2_

**FIGURE 3:**
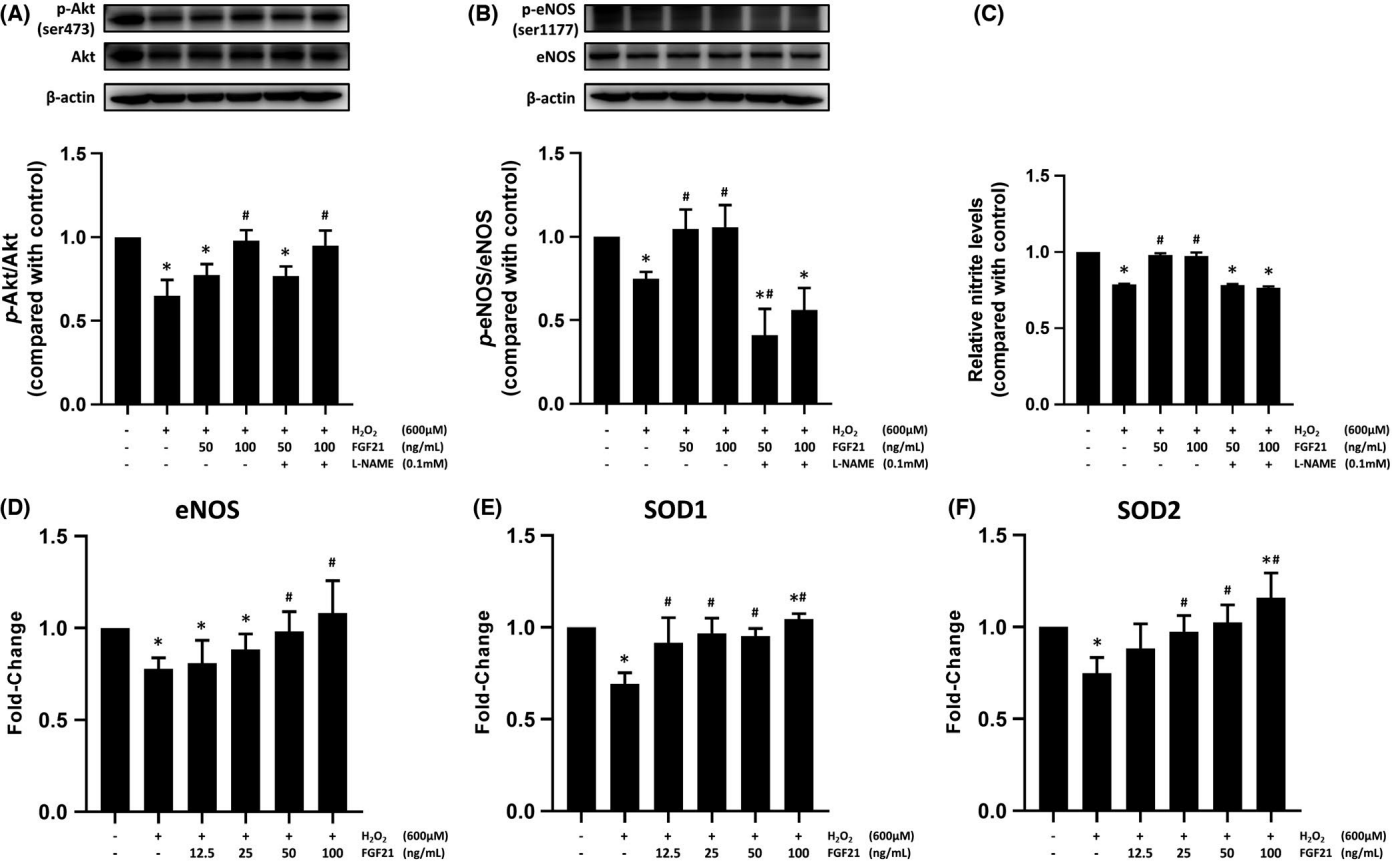
FGF21 mediated the phosphorylation of eNOS and AKT. EPCs were treated with the indicated concentration of FGF21 for 12 h. After FGF21 treatment, the cells were stimulated with 600 µM H_2_O_2_ for 30 min. (A) Phosphorylation of AKT and (B) eNOS was analysed using Western blotting. (C) NO levels were analysed with a Nitric Oxide Colorimetric Assay Kit. Total RNA was extracted from EPCs, and the levels of (D) eNOS, (E) SOD1 and (F) SOD2 were assessed by real‐time PCR. These results are expressed as the mean ± SEM of 5 separate experiments run in triplicate (*p < 0.01 vs. control, #*p* < 0.01 vs. H_2_O_2_ only)

The Akt/eNOS/NO pathway is an important cellular signalling pathway for the migration, angiogenesis and proliferation of EPCs. Previous study showed that FGF21 improves the proliferation and migration of HUVECs via the PI3K/Akt/eNOS pathway.^20^ We investigated the effects of FGF21 on eNOS and Akt expression levels. Compared with the control group, H_2_O_2_ significantly decreased the *p*‐Akt level, and pretreatment with FGF21 significantly attenuated the H_2_O_2_‐mediated suppression of *p*‐Akt expression (Figure 3A). Similarly, H_2_O_2_ significantly decreased the *p*‐eNOS level, and pretreatment with FGF21 attenuated the H_2_O_2_‐mediated suppression of eNOS phosphorylation (Figure 3B). Moreover, treatment of L‐NAME ameliorated the level of p‐eNOS raised by FGF21. These results demonstrate that FGF21 reversed the H_2_O_2_‐reduced expression levels of Akt and eNOS. eNOS is activated in ECs by Akt‐dependent phosphorylation, leading to NO production. H_2_O_2_ inhibited NO production in EPCs, and pretreatment with FGF21 significantly alleviated the reduction in NO production (Figure 3C). To evaluate the expression levels of eNOS, SOD1 and SOD2 in EPCs, the mRNA expression as measured by real‐time PCR. H_2_O_2_ decreased the eNOS, SOD1 and SOD2 mRNA expression, and treatment of FGF21 reversed these effects. (Figure 3D–F).

### 3.6 FGF21 reduced TC and blood glucose levels in ApoE‐KO mice fed a HFD

**FIGURE 4:**
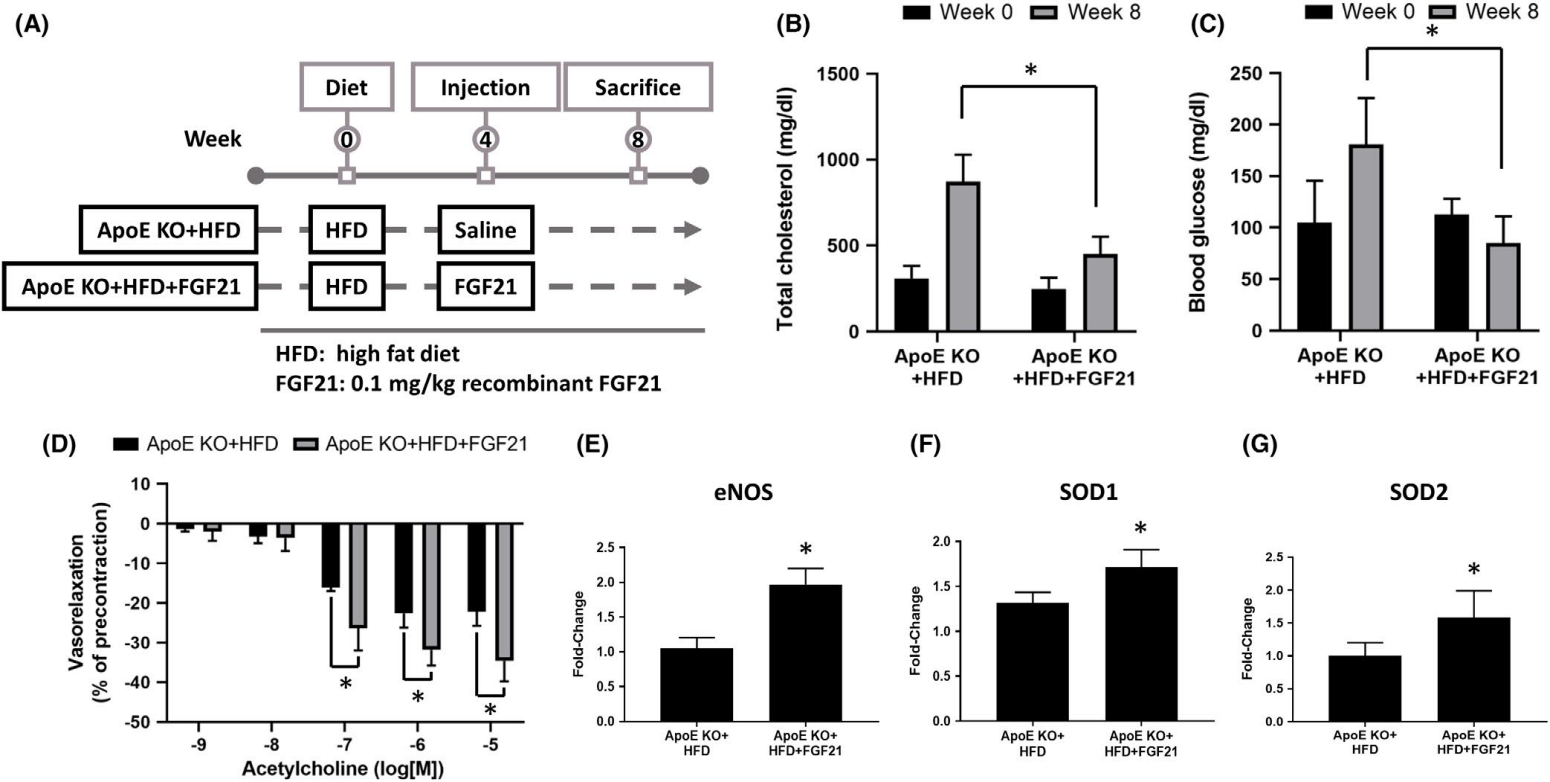
FGF21 ameliorated the HFD‐induced alterations in cholesterol and glucose levels, improved vascular function and increased the levels of eNOS, SOD1 and SOD2. (A) Mice were divided into two groups and fed a HFD for eight weeks. After feeding for 4 weeks, the mice were given 0.1 mg/kg recombinant FGF21 daily by intraperitoneal injection until sacrifice. After sacrifice, the serum was collected, and (B) TC and (C) blood glucose concentrations were measured with an Automated Clinical Chemistry Analyzer. (D) The aorta was cut into 4 mm pieces, and relaxation ability was measured with a force‐displacement transducer. Total RNA was extracted from the aorta, and the levels of (E) eNOS, (F) SOD1 and (G) SOD2 were assessed by real‐time PCR. The results are expressed as the mean ± SEM (*n* = 7, each group) (*p < 0.01 vs. HFD only). One‐way ANOVA followed by Scheffe's multiple comparison post hoc test

We fed ApoE‐KO mice a HFD for eight weeks. After four weeks of feeding, recombinant FGF21 was given daily by intraperitoneal injection (Figure 4A). Serum was collected at the end of the feeding period, and the TC, TG and blood glucose concentrations were measured. The serum TC and blood glucose levels were significantly decreased in ApoE‐KO + HFD + FGF21 mice compared with ApoE‐KO + HFD mice (*p* < 0.001; Figure 4B, C). These data show that FGF21 ameliorated HFD‐induced abnormalities in chemical parameters.

### 3.7 FGF21 improved vasodilation function in ApoE‐KO mice fed a HFD

To investigate the effects of FGF21 on HFD‐induced impairment of endothelial function, we conducted an aortic ring relaxation test. The descending thoracic aorta was isolated from the experimental animals, and vascular reactivity was measured. As shown in Figure 4D, under treatment with acetylcholine, the aortas of FGF21‐treated mice exhibited increased relaxation compared with those of HFD‐fed control ApoE‐KO mice.

### 3.8 FGF21 reversed the expression of eNOS and antioxidant‐related proteins in ApoE‐KO mice

Accumulating evidence indicates that NO is a signalling molecule involved in many physiological and pathological processes and is the main endothelium‐derived relaxation factor. NO is a biologically active unstable radical that is synthesized in vascular endothelial cells by eNOS. Decreased NO bioavailability has been proposed as one of the determinants of vascular damage. To evaluate the mRNA expression levels of eNOS, aortas were isolated from mice, and then, the mRNA expression of eNOS was measured by real‐time PCR. eNOS mRNA expression was increased after FGF21 treatment in ApoE‐KO mice. (Figure 4E). ROS cause endothelial dysfunction and vascular remodelling, and SOD is an antioxidant that exerts effects against superoxides. Reduced SOD activity is associated with increased vascular oxidative stress. We evaluated the mRNA expression levels of SOD1 and SOD2 of FGF21 in ApoE‐KO mice. Interestingly, the mRNA expression of SOD1 and SOD2 was increased in ApoE‐KO + HFD + FGF21 mice (Figure 4F, G).

## 4 DISCUSSION

FGF21 is a member of the FGF family. The FGF family is essential for regulating cell growth, metabolism and differentiation.^21^ In contrast to other members of the FGF family, FGF21 exerts its effects in an endocrine manner.^22^ FGF21 is predominantly produced in the liver, but it is also expressed in adipose tissue,^23^ pancreatic islets,^24^ skeletal muscle,^25^ the hypothalamus^26^ and cardiac endothelial cells.^27^ FGF21 is activated by binding to FGFRs complexed with the essential co‐receptor β‐klotho.^18^ Increased plasma levels of FGF21 were found to be associated with T2DM,^12^ obesity,^13^ metabolic syndrome^14^ and renal dysfunction.^28^ FGF21 treatment alleviates H_2_O_2_‐induced apoptosis and cytotoxicity in HUVECs.^17^ Moreover, a decrease in EPC number and impairment of EPC function have been observed in many chronic diseases, such as diabetes,^29^ hypertension^30^ and chronic kidney disease.^28^

In the present study, we showed that FGF21 regulated TC, TG and blood glucose levels, improved vascular function, increased the expression levels of eNOS, SOD1 and SOD2 in HFD‐fed mice. In addition, we found that EPCs expressed the β‐klotho protein, which might promote the biological effects of FGF21. FGF21 decreases TC levels through suppression of hepatic sterol regulatory element‐binding protein 2 (Srebp‐2) and augmentation of cholesterol efflux, possibly by increasing ABCG5/8 expression in ApoE and FGF21 double KO mice,^16^ and prevents increases in blood glucose levels in a type 1 diabetes mouse model.^31^ This finding is consistent with our current results. The administration of FGF21 significantly reduced cholesterol and blood glucose levels in ApoE‐KO mice. Substantial clinical and experimental evidence has suggested that both hyperglycaemia and dyslipidaemia contribute to increased production of ROS.^32^ Excessive production of ROS leads to endothelial dysfunction and reduced NO bioactivity.^33^ Hypercholesterolemia increases ROS production and endothelial dysfunction in ApoE‐KO mice.^34^ Of note, daily injection of FGF21 promoted endothelium‐dependent vasoreactivity by improving sensitivity to Ach‐induced vascular relaxation and increasing the mRNA expression of eNOS and anti‐oxidative genes, including SOD1 and SOD2.

Increased FGF21 levels have been reported to be associated with atherosclerosis and CAD.^35^ However, the mechanism underlying the protective effect of FGF21 on the cardiovascular system remains to be determined. Some studies have indicated that FGF21 treatment relieves H_2_O_2_‐induced apoptosis and cytotoxicity in HUVECs.^17^ Although FGF21 improves HUVEC functions, the potential effect of FGF21 on EPCs remains unknown. We showed that the administration of FGF21 improved the viability, migration and tube formation ability of EPCs in the presence of a high level of ROS. Oxidative stress is well known to affect EPC survival. EPCs were exposed to H_2_O_2_, which induces ROS production. Pretreatment with FGF21 reduced ROS production in EPCs under H_2_O_2_ exposure. However, the mechanism underlying the antioxidant effect of FGF21 on EPCs remains unclear. FGF21 exerts an antioxidant effect against oxidative stress in the heart through the AMPK‐induced antioxidative (Akt–GSK3β–Fyn–Nrf2) pathway and promotes the antioxidant gene expression of uncoupling protein (Ucp)2, Ucp3 and SOD2.^36^ The SOD system, which defends against ROS, plays an important role in endothelial dysfunction and is present in vascular tissue.^37^ Overexpression of SOD in ApoE‐KO mice alleviates atherosclerotic lesions in the early stages.^38^ We found that FGF21 exerted antioxidant effects in ApoE‐KO mice by elevating the expression of SODs.

β‐klotho is a cofactor that is required for FGF21 binding to FGFR1. Lack of Klotho in murine models causes accelerated aging syndrome, atherosclerosis, vascular calcifications,^39^ defects in angiogenesis and endothelial dysfunction.^40^ Recent studies have shown that Klotho protects the vascular system, including endothelial homeostasis and vascular functionality,^40^ and that loss of klotho contributes to endothelial dysfunction and vascular calcifications.^40^ Therefore, inhibition of β‐klotho expression affects the ability of FGF21 to activate the intracellular signalling pathway. However, there is limited data regarding β‐klotho expression in EPCs. We found that β‐klotho was expressed in EPCs, suggesting that FGF21 can exert a direct effect on EPCs.

**FIGURE 5:**
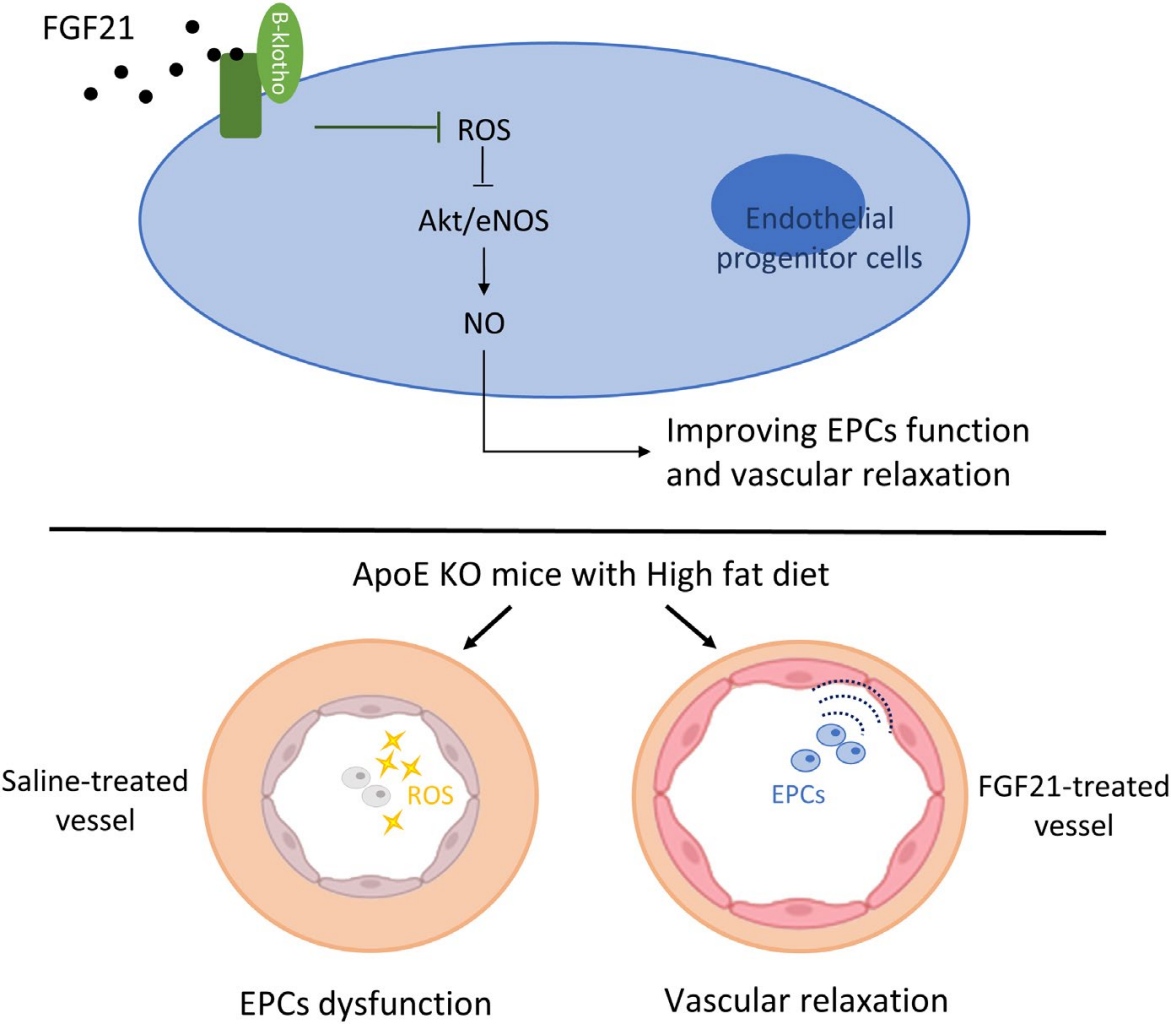
Proposed scheme of the potential protective effects of FGF21 on EPCs in response to high oxidative stress conditions. In summary, FGF21 improves EPC functions via the Akt/eNOS/NO pathway and reverses endothelial dysfunction under oxidative stress

The Akt/eNOS signalling pathway is essential for mediating EPC survival and function.^41^ Activation of the Akt/eNOS signalling pathway in EPCs increases the cell number, mobilization, NO production and vasodilation.^5^ We found that FGF21 increased the phosphorylation of Akt at Ser473 and the phosphorylation of eNOS at Ser1177 under H_2_O_2_ exposure. Recent evidence suggests that NO is important for maintaining EPC function.^42^ NO can induce the differentiation of EPCs into mature ECs or stimulate EPC mobilization from the bone marrow (BM) to the peripheral circulation.^7^ To confirm whether FGF21 improved EPC function through the Akt/eNOS/NO signalling pathway, we used an eNOS inhibitor (L‐NAME) to block this specific pathway. The beneficial effects of FGF21 on EPCs were inhibited after L‐NAME treatment. Our studies indicated that increased NO production improved the migration and tube formation ability of EPCs (Figure 5).

## 5 CONCLUSIONS

In summary, our experimental findings showed that FGF21 directly modulated EPCs. Administration of FGF21 improved the function of oxidative stress‐exposed EPCs by activating the Akt/eNOS/NO signalling pathway. In addition, treatment with FGF21 improved the metabolism of lipids and glucose and further restored endothelial function. This study suggests that FGF21 might be a novel molecular target for metabolic syndromes.

## CONFLICT OF INTEREST

The authors confirm that there are no conflicts of interest.

## AUTHOR CONTRIBUTIONS

**Wen‐Pin Huang:** Conceptualization (lead); Data curation (equal); Formal analysis (equal); Investigation (lead); Validation (equal); Writing – original draft (equal); Writing – review & editing (equal). **Chi‐Yu Chen:** Conceptualization (lead); Data curation (lead); Formal analysis (lead); Investigation (lead); Methodology (lead); Validation (lead); Writing – original draft (lead); Writing – review & editing (equal). **Tzu‐Wen Lin:** Data curation (supporting); Formal analysis (supporting); Investigation (supporting); Methodology (supporting); Validation (supporting); Writing – original draft (supporting); Writing – review & editing (supporting). **Chin‐Sung Kuo:** Formal analysis (supporting); Methodology (supporting); Resources (supporting); Writing – review & editing (supporting). **Hsin‐Lei Huang:** Formal analysis (supporting); Methodology (supporting); Project administration (equal); Validation (equal); Writing – review & editing (equal). **Po‐Hsun Huang:** Conceptualization (equal); Funding acquisition (lead); Project administration (lead); Resources (lead); Supervision (lead); Writing – review & editing (lead). **Shing‐Jong Lin:** Project administration (supporting); Supervision (supporting); Writing – review & editing (supporting).

## Supporting information

Fig S1

## Data Availability

The datasets used and/or analysed during the current study are available from the corresponding author on reasonable request.
