# Supplementary figures and images for: Fibroblast growth factor 21 reverses high‐fat diet‐induced impairment of vascular function via the anti‐oxidative pathway in ApoE knockout mice
Source: J Cell Mol Med. 2022 Mar 20;26(8):2451–61. doi: 10.1111/jcmm.17273 (PMC8995458; doi:10.1111/jcmm.17273)

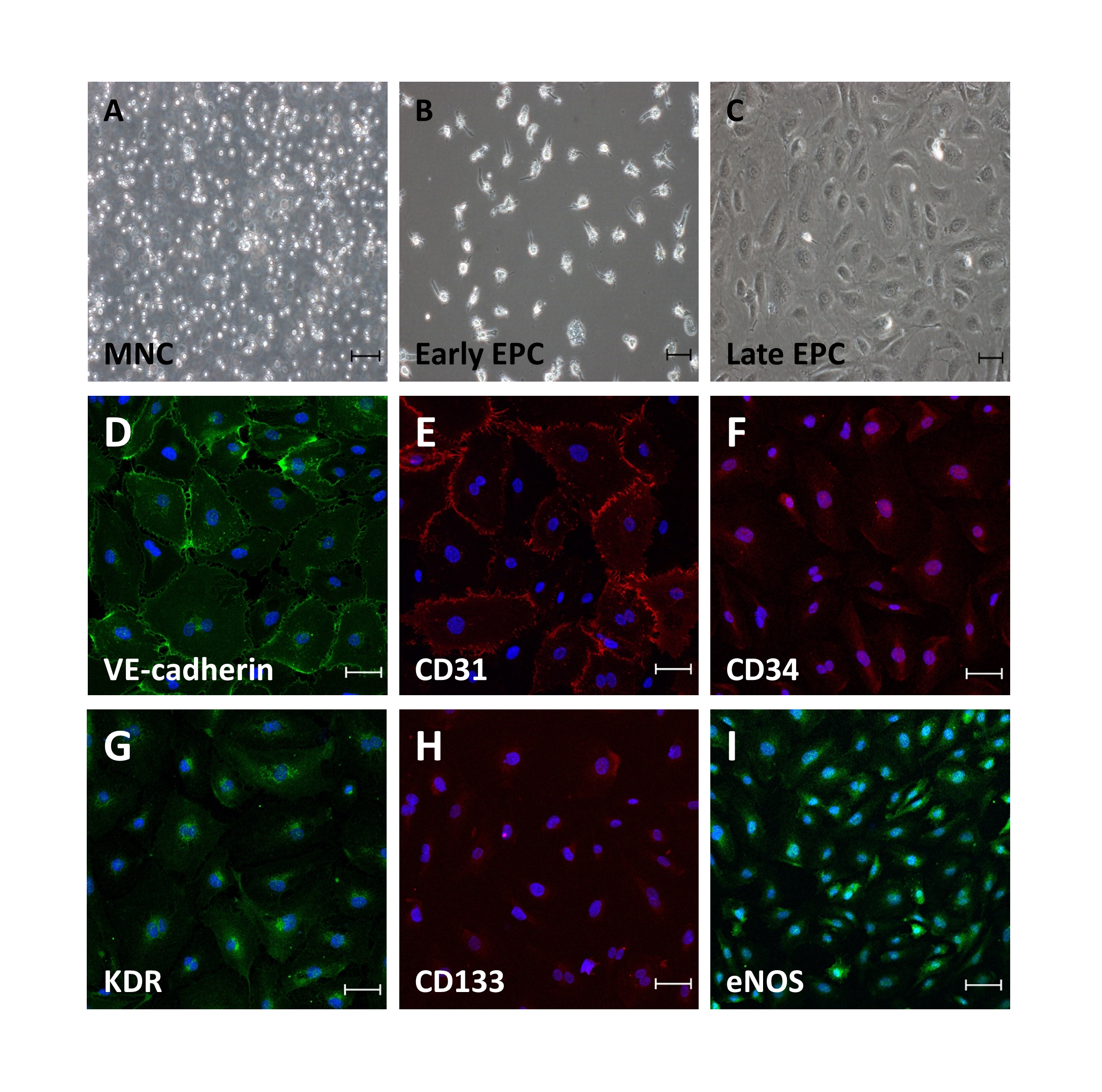

Supplement: Supplementary file 1 — Fig S1 [file JCMM-26-2451-s001.tif]
